# Iron Is the Active Site in Nickel/Iron Water Oxidation Electrocatalysts

**DOI:** 10.3390/molecules23040903

**Published:** 2018-04-14

**Authors:** Bryan M. Hunter, Jay R. Winkler, Harry B. Gray

**Affiliations:** Beckman Institute, California Institute of Technology, Pasadena, CA 91125, USA; bhunter@caltech.edu

**Keywords:** oxygen evolution reaction, layered double hydroxide, electrocatalyst

## Abstract

Efficient catalysis of the oxygen-evolution half-reaction (OER) is a pivotal requirement for the development of practical solar-driven water splitting devices. Heterogeneous OER electrocatalysts containing first-row transition metal oxides and hydroxides have attracted considerable recent interest, owing in part to the high abundance and low cost of starting materials. Among the best performing OER electrocatalysts are mixed Fe/Ni layered double hydroxides (LDH). A review of the available experimental data leads to the conclusion that iron is the active site for [NiFe]-LDH-catalyzed alkaline water oxidation.

## 1. Background

The urgency to develop new technologies that harness energy and natural feedstocks in a sustainable fashion has never been more apparent. With global power consumption growing at an exponential rate, only one resource is truly capable of powering the planet: the sun. Sunlight is reliable, clean, and free.

**Scheme 1:**
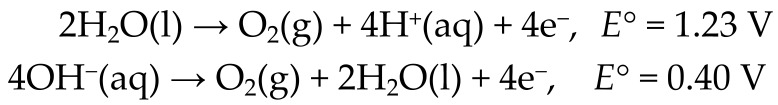
Oxygen Evolution Half-Reactions (OER).

The long-standing goal of converting radiant solar energy into chemical fuels [1,2,3] has received renewed attention in the past decade [4,5,6]. Solar-driven water splitting to H_2_ and O_2_ remains the preeminent objective. Although considerable progress has been made in the development of scalable devices, there is a clear need for robust earth-abundant materials that can catalyze the oxygen evolution half-reaction (OER) in both acidic and alkaline media (Scheme 1) [5,7].

Heterogeneous water oxidation electrocatalysts containing first-row transition metal oxides and hydroxides have been extensively explored, owing in part to the high abundance and low cost of starting materials [7]. FeOOH is the most active single first-row transition metal water oxidation catalyst [8], and iron impurities on gold also have shown excellent performance [9]. Among the best OER catalysts, however, are mixed Fe/Ni layered double hydroxides (LDH). The M(OH)_2_ LDH hexagonal crystal lattice is a brucite structure consisting of layers of edge-shared M(OH)_6_ octahedra [10]. The interlayer spacing is defined by the crystallographic *c*-axis length. In anhydrous materials, *c* is about 4.5 Å; the layers expand when H_2_O intercalates leading to *c* ~ 7–8 Å. Partial incorporation of M^3+^ ions in place of M^2+^ is accompanied by intercalation of anions between the layers to balance the extra positive charge, producing large interlayer spacings (*c* ~ 7–8 Å). The most active [NiFe]-LDH OER catalysts have a Ni:Fe ratio of ~3:1. Before examining the role of Fe in these materials, it is helpful first to review the electrochemical and electrocatalytic properties of the parent LDH: Ni(OH)_2_.

## 2. Nickel Hydroxide Electrodes

**Figure 1:**
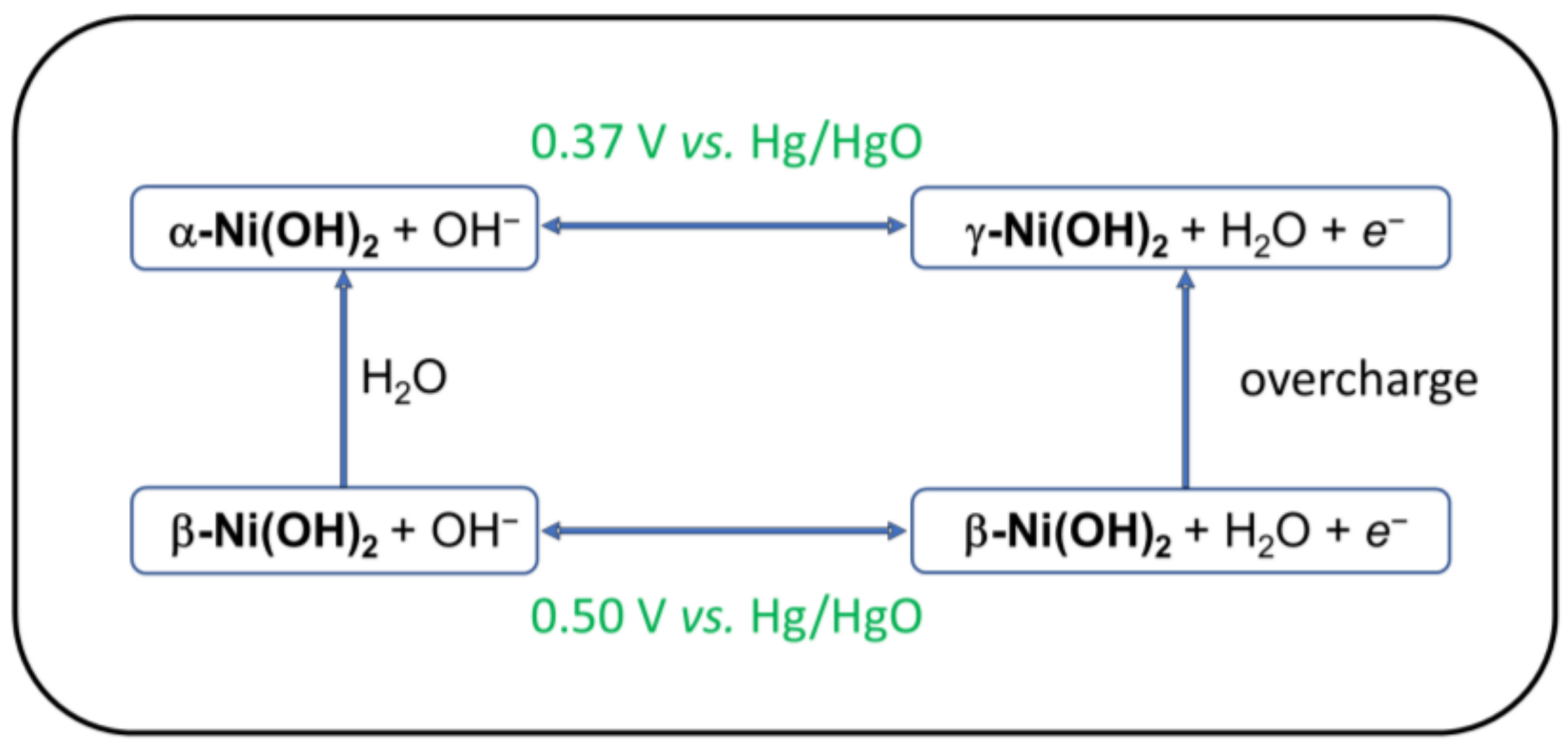
Bode cycle indicating electrochemical phase changes for Ni(OH)_2_.

The important role of Ni-hydroxides in battery and electrolyzer technology has stimulated a vast amount of research into the structural, chemical, and electrochemical properties of this material [10,11]. Most discussions of Ni(OH)_2_ electrochemistry begin with a square scheme known as the Bode cycle (Figure 1) [12]. This cycle identifies two Ni(II)-hydroxides (α-Ni(OH)_2_, β-Ni(OH)_2_) and two oxidized forms defined as γ-Ni(O)(OH), β-Ni(O)(OH). The formal potentials for the two couples are as follows: *E*°(γ-Ni(O)(OH)/(α-Ni(OH)_2_) = 0.37 V vs. Hg/HgO; *E*°(β-Ni(O)(OH)/(β-Ni(OH)_2_) = 0.50 V [13]. The primary structural distinction between hexagonal crystals of α-Ni(OH)_2_ and β-Ni(OH)_2_ is the interlayer spacing. The layers in α-Ni(OH)_2_·*x*H_2_O (0.4 ≤ *x* ≤ 0.7) are intercalated with water molecules, leading to *c* ~ 8 Å, whereas *c* ~ 4.6 Å in anhydrous β-Ni(OH)_2_ [10].

**Scheme 2:**
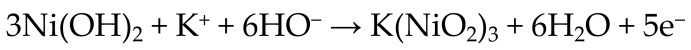
Proposed 1.67 e^−^ per Nickel Atom Couple [19].

Although the Ni(O)(OH) materials formally contain Ni(III), a large amount of early research suggested that the average oxidation state in γ-Ni(O)(OH) was greater than +3. On the basis of electrochemical and iodometric measurements, Corrigan concluded that oxidation of Ni(OH)_2_ produces a material with an average oxidation state of +3.67 [14,15,16]. Notably, these studies did not take into account the iron impurities that were later found to contaminate most Ni(OH)_2_ samples (*vide infra*) [14,17,18]. On the basis of the calculated oxidation state change, the couple in Scheme 2 was proposed [16,19]. The redox process in Scheme 2 corresponds to a structural rearrangement of α-Ni(OH)_2_ to γ-Ni(O)(OH) [10]. This interpretation was further supported by X-ray spectroscopic measurements that indicated average oxidation states of +3 in β-Ni(O)(OH) and ≥+3.5 in γ-Ni(O)(OH) [20,21].

Not all of the data are consistent with this simple interpretation. In particular, the charge and discharge cycles are not symmetric, indicating that γ-Ni(O)(OH) represents a major structural rearrangement in a portion of the material. Under galvanostatic control, ca. 1 electron per nickel center is transferred to the electrode at the first plateau (0.4 V vs. Hg/HgO), then the potential increases to 0.6 V vs. Hg/HgO [16]. This behavior, often called “overcharging,” indicates that the single “pre-wave” in LDH materials is direct oxidation to Ni(III). 

Furthermore, under cathodic cycling of the electrode, only 1.12–1.32 electrons per Ni atom can be delivered. Following cycling, the electrode remains black, never reverting to the almost colorless Ni(OH)_2_ [16]. Although Corrigan attributes the asymmetric cycling to the insulating behavior of Ni(OH)_2_ preventing some NiOOH from being reduced, there is nevertheless irreversible restructuring of the material. This restructuring, while of importance to applications involving battery materials, apparently does not play a role in OER catalysis (the electrode does not visibly darken during in situ anodization in nonaqueous media) [22].

## 3. Iron Incorporation into Ni(OH)_2_

Catalysis of water oxidation by nickel hydroxide has been studied since the 1960s [11,12]. However, is nickel the active site? Not likely. Iron, a potentially better catalytic metal, is present in most Ni(OH)_2_ samples. What is more, the intentional incorporation of iron into Ni-LDH structures dramatically enhances OER activity. This behavior has been attributed to enhanced Ni catalysis due to the Lewis acidity of Fe(III) [23]. However, Fe(III) is more than a Lewis acid; these redox-active ions in the Ni-LDH lattice cause a charge imbalance in the M(OH)_6_ layers that is compensated by interlayer anions [24]. For [NiFe]-LDH in strongly alkaline solution, that anion is almost always CO_3_^2−^ [25]. Boettcher suggested that layered structures are critically important for highly efficient water oxidation catalysis [26]. However, exfoliation of iron-containing nickel LDHs into individual “sheets” led to improved performance [27].

Corrigan first recognized the influence of adventitious (as well as intentional) iron incorporation into nickel oxide electrodes [14]. In one 1987 study, effects were apparent even at 0.01% iron loading. In films of high iron content (10%), major changes in Tafel slopes were observed; notably, slopes were lowered from ca. 70 mV/decade (pure nickel) to 25 mV/decade. Those who swear by Tafel slopes would conclude that incorporation of iron fundamentally changed the nature of the catalytic reaction. Perhaps of greater relevance is that a > 200-mV decrease in overpotential accompanied the Tafel slope change. Given these findings, it is shocking that most subsequent work largely ignored the role of incidental iron incorporation.

More than twenty-five years after Corrigan’s study, Boettcher and coworkers systematically characterized nickel(II) hydroxide films with added iron [18]. They found that incorporation of iron increased film conductivity by a factor of 30, but that this finding alone was insufficient to explain the improved activity. Furthermore, they observed that iron migrates into LDH materials with alarming ease. Upon redox cycling in the highest-purity KOH electrolyte available, these investigators found a 95:5 Ni:Fe ratio after just 12 min. Interestingly, iron incorporation in CoOOH materials shows similar effects [28].

In a subsequent study, Boettcher’s group reported that the Ni(III)/Ni(II) “pre-wave” did not correlate with water oxidation activity, further calling into question the role of nickel in iron-containing catalysts. Importantly, the magnified effect of Fe in spin-cast films is unique; other metals, notably La, Mn, Ce, and Ti, do not provide long-term improvement [29]. 

In other work, Boettcher was able to incorporate iron directly from solution at edge and defect sites, showing that these specific sites are directly related to OER performance, while bulk iron substitution only influences the nickel charging reaction [17]. The observation that iron affects nickel charging but not the overpotential for water oxidation is strong evidence that nickel-only materials cannot oxidize water at potentials that are active for [NiFe]-LDH catalysts. Indeed, the OER catalytic wave in highly Fe-free Ni(OH)_2_ electrodes occurs at potentials at least 200 mV more positive than for electrodes containing even a trace of Fe. Moreover, the catalytic wave shifts further anodically upon Fe-free Ni(OH)_2_ electrode cycling, behavior unlike that of electrodes in the presence of trace Fe [18]. The role of iron is not simply to modulate the oxidizing capability of nickel. Iron is the one and only active site. 

## 4. Nickel Is Not Oxidized to Ni(IV)

It is our view that the early literature on nickel oxide electrodes in aqueous alkaline solution is largely irrelevant to the discussion of key steps in [NiFe]-LDH catalyzed OER. Incorporation of Fe(III) into the Ni(OH)_2_ lattice requires the intercalation of anions between M(OH)_6_ layers, preventing formation of the β-Ni(OH)_2_ (brucite) structure and interfering with the Bode cycle. Oxidation of Ni in this material might be expected to produce γ-Ni(O)(OH), which, in spite of its chemical formula, is thought to be primarily Ni(IV). Boettcher’s work demonstrates that as the proportion of Fe(III) in the lattice increases, the NI(II) oxidation wave shifts anodically: in a 75:25 Ni:Fe material, the Ni(II) oxidation wave cannot be observed prior to the onset of catalytic OER current [18,30]. Boettcher rationalized this shift on the basis of the increased positive charge in the M(OH)_6_ lattice. Moreover, his work with Fe-free Ni(OH)_2_ indicated that its overpotential for OER is extremely large, far greater than that of a material containing Fe. The poorer performance of the nickel-only material also revealed previously unseen features, including a new oxidation at ~0.6 V versus Hg/HgO, which is past the onset of water oxidation in iron-containing samples. The similarity of these potentials and those found galvanostatically is worth noting. It is clear that the presence of iron makes the oxidation of nickel more difficult, precluding the formation of Ni(IV).

It is likely that nickel electrochemistry in [NiFe]-LDH is similar to that in [NiAl]-LDH [31]. Interestingly, Qiu and Villemure found that charging [NiAl]-LDH in 0.2 M KOH at 0.6 V vs. SCE (~0.74 vs. Hg/HgO) produces Ni(III). This material can be reduced readily back to Ni(II). Charging at 0.8 V versus SCE, these investigators found that nickel was in the +3.6 oxidation state, and that the reaction was reversible. Higher potentials produced some Ni(IV), but reduction in this case only gave Ni(+2.6) [32]. Although nickel reaches a high oxidation state in these materials, the potentials are much higher than OER catalyzed by [NiFe]-LDH nanosheets. OER in the nickel/aluminum materials was observed at 580 mV vs. Hg/HgO in 7 M KOH [33].

## 5. The Role of High-Oxidation-State Iron in OER

The potentials for oxidation of the metal centers in [NiFe]-LDH are moderated by accompanying deprotonation of H_2_O and OH^−^ ligands; at high states of electrode charge, these deprotonation reactions will produce terminal oxo ligands. Oxo ligands are far more likely to be found on high-oxidation-state iron [Fe(IV), Fe(V), Fe(VI)] than high-oxidation-state nickel [Ni(IV)] at potentials relevant to water oxidation in alkaline media. The stabilities of metal–oxo complexes decrease across the periodic table, owing to the addition of *d* electrons to π* orbitals (there is an “oxo wall” between Fe-Ru-Os and Co-Rh-Ir in the periodic table) [34]. Relevant to this discussion, there are no well-characterized Ni(IV) oxos, as expected (nickel is on the wrong side of the wall).

Pourbaix (potential-pH) diagrams for nickel and iron illustrate that at pH 14 the Fe(VI)/Fe(III) couple is virtually identical with the γ-Ni(O)(OH)/Ni(OH)_2_ couple [35,36]. Moreover, tetraoxoferrate(VI) does not to react with Ni(III), suggesting that the Ni(III)/Ni(II) couple is above the Fe(VI)/Fe(V) couple [37]. For these reasons, iron in LDH materials is oxidized to high (>IV) oxidation states before nickel is oxidized to Ni(IV).

**Figure 2:**
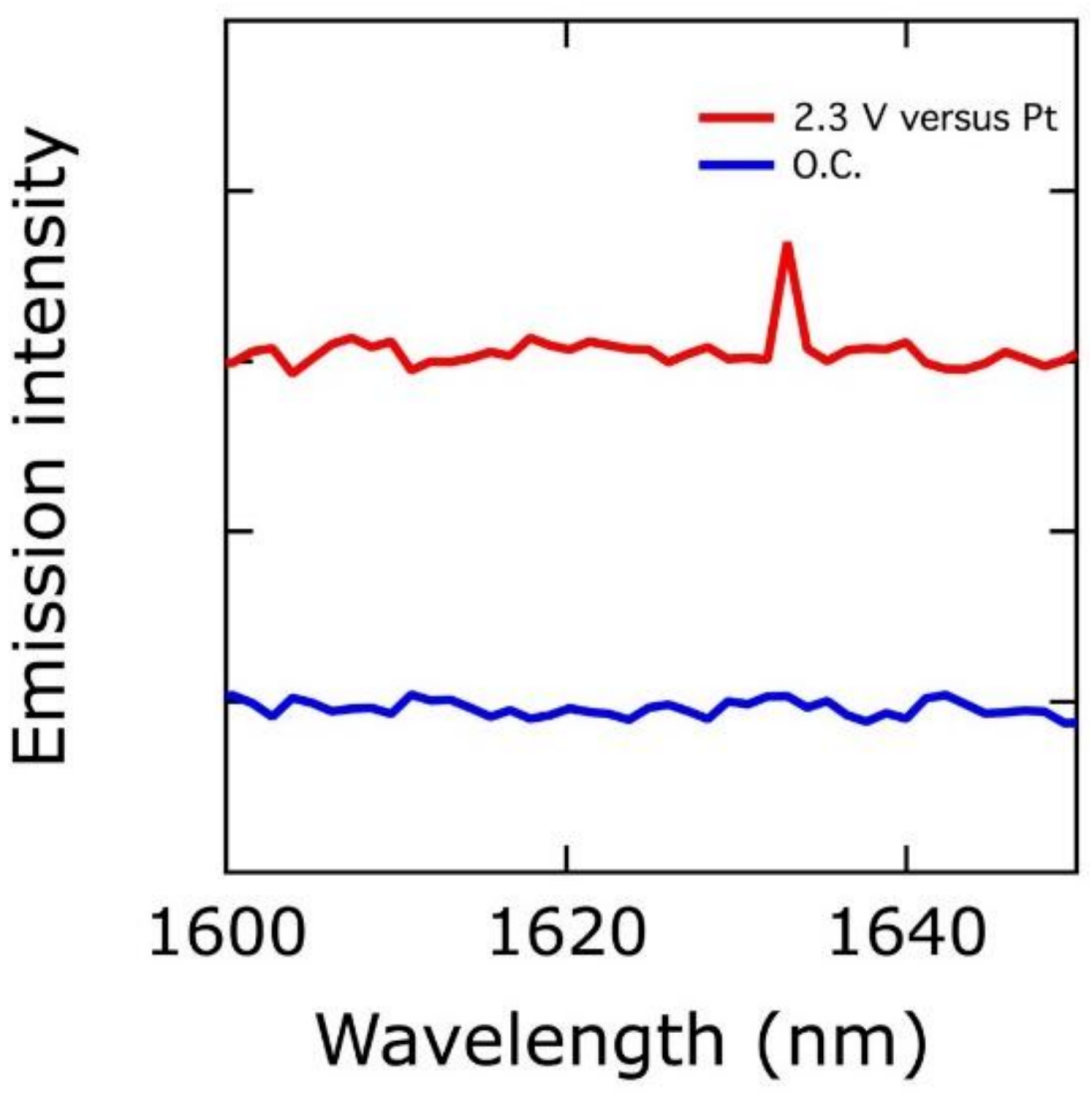
In red: near-IR luminescence spectrum (applied potential, 2.3 V vs. Pt) of [NiFe]-LDH. In blue: reference spectrum of the material taken at the open circuit potential (O.C.).

We recently reported in situ infrared, luminescence, and Mössbauer spectra consistent with the presence of cis-dioxo Fe(VI) in [NiFe]-LDH (the ground state of cis-dioxo Fe(VI) is a spin-triplet; the signature spin-flip excited-singlet to triplet emission is shown in Figure 2) [22]. These spectroscopic features were present in a minority (ca. 3%) of iron sites, as would be expected for localization at corner sites in LDH nanosheets. Kinetics modeling revealed that a measurable population of reactive intermediates could be produced by limiting access to substrate (H_2_O and HO^−^) [22]. Taken together, the evidence strongly indicates that Fe(VI) plays a key role in water oxidation. We were not the first to come to this conclusion, as Lyons and Brandon previously suggested that a high-oxidation-state iron species might be the active site [38].

*In-operando* XAS studies of these materials indicate that, upon polarization of a 75:25 Ni:Fe material, the Ni K-edge and pre-edge shift to higher energies, consistent with an increase in the Ni oxidation state [39]. The Fe-edge, however, does not exhibit a potential-induced shift. EXAFS analysis of polarized electrodes indicates that both Ni–O and Fe–O distances decrease to ~1.9 Å (from *d*(Ni–O) = 2.06 and *d*(Fe–O) = 2.01 Å in the unpolarized electrode). The Ni–O bond shortening is consistent with the increase in Ni oxidation state, but the Fe bond contraction is difficult to rationalize if the Fe oxidation state remains unchanged. A possible explanation for this behavior is that the lattice contraction that accompanies Ni oxidation imposes a stronger ligand field on the Fe(III) centers, thereby inducing crossover from a high-spin electronic structure to a low-spin state [Fe–O distances of ~1.9 Å are consistent with low-spin Fe(III)] [40]. Synergistic coupling of Ni(II,III) with Fe(III) spin states could account for the anodic shift in *E*°(γ-Ni(O)(OH)/(α-Ni(OH)_2_) as the concentration of Fe increases [18,30]. 

Why, then, is there no observable shift in the K-edge for iron? The kinetics of the process being observed also is of critical importance. The failure to observe high-oxidation-state Fe *in situ* may simply be a consequence of the high OER activity of the material: rapid reaction with HO^−^ could deplete the population of Fe(VI) as rapidly as it is formed, leading to steady-state concentrations too small to be detected by techniques such as XAS [41]. For this reason, we performed our studies at low substrate concentrations, which sufficiently slowed the relative rate of consumption of intermediates. 

Taken together, the experimental facts—the variation in water oxidation activity upon the incorporation of iron, the relative difficulty in oxidizing Ni(III) to more reactive species, and the direct evidence of high-oxidation-state iron in [NiFe]-LDH materials—lead to the firm conclusion that iron is the active site for [NiFe]-LDH-catalyzed alkaline water oxidation.
